# Achenbach Syndrome: A Case Report

**DOI:** 10.5811/cpcem.1917

**Published:** 2024-11-09

**Authors:** Michelle Chen, Debby Yanes, Pallavi Manvar-Singh, Christopher Iamonaco, Christopher John Jerome, Azhar Supariwala

**Affiliations:** * Northwell Health, Southshore University Hospital, Department of Emergency Medicine, Bay Shore, New York; † Northwell Health, Southshore University Hospital, Department of Vascular Surgery, Bay Shore, New York; ‡ Northwell Health, Syosset Hospital, Department of Emergency Medicine, Syosset, New York; § Northwell Health, Southshore University Hospital, Department of Orthopedics, Bay Shore, New York; || Northwell Health, Southshore University Hospital, Department of Cardiology, Bay Shore, New York

**Keywords:** Achenbach syndrome, finger discoloration, case report

## Abstract

**Introduction:**

Achenbach syndrome is a rare, benign condition characterized by painful discoloration of a finger. Recognition of this syndrome prevents unnecessary costly workup and risky interventions.

**Case Report:**

A healthy, 54-year-old female was transferred to our emergency department (ED) from a community ED for vascular evaluation of discoloration and numbness to her finger. After extensive workup, medical intervention, and consultation with multiple specialists, she was diagnosed with Achenbach syndrome.

**Conclusion:**

Emergency physicians may practice good healthcare stewardship and limit invasive, potentially harmful, and expensive workup by reassuring patients of the benign nature of this condition.

## INTRODUCTION

Achenbach syndrome is a relatively rare, likely under-reported, benign, painful discoloration of a finger that is self-resolving. Despite being first reported in 1958 by Dr. Walter Achenbach, this condition has primarily been documented in case reports and small case series. Most commonly, patients are middle-aged females presenting with atraumatic ecchymosis of the volar aspect of a single digit without any identifiable risk factors such as smoking, coagulopathies, trauma, drug use, and rheumatologic disorders.^1^–^3^ The etiology is unknown and does not appear to have permanent sequelae.^1^ Despite the benign course of this condition, the presentation of Achenbach syndrome is often distressing to patients and clinicians alike. As this is a little-known syndrome, patients are at times subject to unnecessary workup leading to increased anxiety, cost, and risks of interventions. Here we present a case of an otherwise healthy female who was transferred to our tertiary care center for higher level of care to evaluate an atraumatic finger with discoloration and numbness, concerning for ischemic etiology.

## CASE REPORT

**Image 1:**
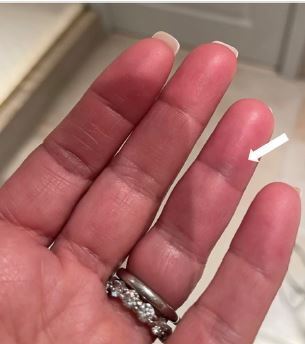
A bluish discoloration resembling a circle noted to the volar aspect of the left fourth digit just distal to the crease of the distal phalanx. Of note, the dark discoloration seen in the second, third, and proximal aspect of the fourthth digit are the result of a shadow from the patient’s phone.

**Image 2:**
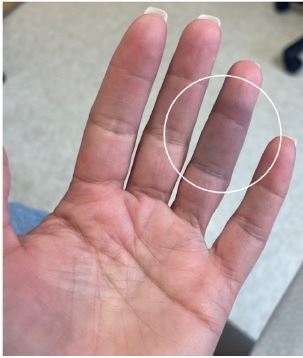
A well-demarcated area of ecchymosis to the volar aspect of the fourth digit on the left encompassing the entire middle phalanx with some extension into the distal phalanx.

A54-year-old White female with no past medical history was transferred to our emergency department (ED) from a community ED in the summer season for further vascular evaluation of the discoloration and numbness to her left fourth digit. The patient reported feeling a sudden sharp pinch in the middle phalanx the prior evening and then noting the development of a blue circle (Image 1). She awoke the next morning to find that her entire middle phalanx appeared bruised with some tenderness to palpation and numbness (Image 2). The patient was a former smoker of nine years and had quit 30 years earlier. She denied prior history of similar events.

On physical exam, her left fourth digit demonstrated a bluish discoloration limited to the volar aspect of her middle phalanx; it was warm to touch, with decreased sensation and slight tenderness to the affected area. The distal phalanx had normal color with good capillary refill. The patient had full range of motion of all digits. The remainder of her left hand appeared within normal limits. Her radial pulses were equal and strong bilaterally.

**Population Health Research Capsule**

What do we already know about this clinical entity?

*Achenbach syndrome, characterized by painful discoloration of a finger, is a rare disorder that is completely benign and self-resolving.*

What makes this presentation of disease reportable?

*Achenbach syndrome often goes unrecognized, leading to costly and potentially harmful workups.*

What is the major learning point?

*Physicians should feel comfortable diagnosing Achenbach syndrome and reassuring patients of its benign nature.*

How might this improve emergency-medicine practice?

*Awareness of this disorder allows emergency physicians to consider it in a differential, diagnose as appropriate, and save the patient time, cost, and anxiety.*

**Image 3:**
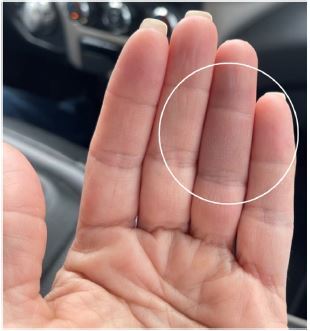
A mostly resolved area of ecchymosis seen at the volar aspect of the fourth digit on the left.

The patient was initially evaluated at a community ED where there was a concern for vascular injury. Prior to transfer to obtain specialty consultation, she underwent an ultrasound arterial duplex of the left upper extremity, which was found to be normal, and she was started on full anticoagulation via heparin drip. The patient was admitted into our hospital and was continued on the heparin drip. She received a transthoracic echo with bubble study, which demonstrated an intact interatrial septum, and she was evaluated by multiple specialists including vascular surgery and cardiology to assess for a cardioembolic source of ischemia and later an orthopedic-hand specialist for other possible diagnoses. All lab work including erythrocyte sedimentation rate (ESR) and C-reactive protein (CRP) were normal. Ultimately, the patient was diagnosed with Achenbach syndrome and discharged home. Her symptoms self-resolved after four days (Image 3).

## DISCUSSION

Achenbach syndrome is a benign and self-resolving condition often presenting as acute discoloration, pain, and numbness of one or more digits. Despite first being reported in 1958, this remains a little-known disease which, on review of current available case reports, often results in unnecessary testing, consultations, and anxiety for patients. The emergent concern in the initial presentation of Achenbach syndrome is acute limb ischemia where thrombosis, embolism, or dissection may lead to sudden arterial occlusion. If this were the case, patients would commonly experience pain, cold skin, pallor, or pulselessness.^4^ However, in Achenbach syndrome, pulses and temperature are intact, and patients tend to present with ecchymosis of just the volar aspect of a digit. Reports indicate a propensity for this syndrome to occur on the volar aspect of the third or fourth digits in women but have also been reported to occur in men and on nearly all other digits, the dorsal aspect of digits, the palm, and even toes.^1^ In our patient, sparing of the distal phalanx was very prominent; this finding may not be the case in all patients with this syndrome.

Patients often undergo vascular studies such as Doppler ultrasonography, echocardiograms, and computed tomography angiography, which are normal.^1^ A report of a patient with Achenbach syndrome who underwent punch biopsy revealed multiple ectatic capillaries in the dermal layer, in addition to some extravasation of red blood cells out of these vessels into the dermis, which would suggest some degree of vessel fragility.^5^ A separate report of a patient who underwent capillaroscopy demonstrated hemorrhages without change in capillary morphology or blood flow, indicating that capillary microhemorrhages can be observed in Achenbach syndrome.^6^

Vasculitis is another differential to consider with this presentation. In vasculitis, symptom resolution generally takes weeks to months, rather than days as in Achenbach syndrome.^1^ There are often notable laboratory features in vasculitis such as elevated ESR, CRP, and antinuclear antibody levels.^5^ In addition, there are frequently other clinical clues to suggest these diagnoses. A strong history of tobacco use may suggest thromboangiitis obliterans, which most commonly manifests as signs and symptoms of limb ischemia in the most distal portions of an extremity and progresses proximally in the setting of persistent tobacco use, often involving multiple digits and extremities.^7^

Exposure to cold temperatures could suggest Raynaud phenomenon or pernio. Classically, patients experiencing Raynaud phenomenon complain of symmetric color changes in multiple digits, generally beginning as white due to tissue ischemia resulting from vasospasm of the arteries, then blue or purple, and finally red as the tissues reperfuse once rewarmed. Pernio occurs after prolonged exposure to temperatures above freezing. Patients develop painful, violaceous papules usually on the dorsal aspect of multiple fingers or toes, without the color change as noted in Raynaud.^8^ Recent stress or anxiety could suggest psychogenic purpura.^9^ In psychogenic purpura, patients develop ecchymoses in various locations, generally on the extremities, that are associated with different prodromes ranging from itchiness to pain or warmth.^10^

In general, Achenbach syndrome involves ecchymosis of a single digit or part of a single digit, which detracts largely from the aforementioned conditions that often involve multiple digits or different bodily locations. This is by no means an exhaustive list of differentials, but just a few to illustrate the importance of a thorough history and physical exam to help differentiate potential causes of this presentation. Additionally, a recent report revealed a possible genetic component to Achenbach syndrome, detailing three cases within two successive generations of middle- to older-aged women.^11^ Obtaining a careful family history may aid clinicians in making this diagnosis. Further evaluation would be useful in elucidating whether these genetic factors may be associated with other pathologies of concern.

In an early systematic review of the medical literature on Achenbach syndrome, Kordzadeh formulated an algorithmic approach for diagnosis and management based upon 12 case reports. Kordzadeh suggests initially assessing pulses in any patient presenting with bluish discoloration, pain, edema, and paresthesia of a digit. If pulses are not palpable, the clinician should work up the patient for acute limb ischemia. If pulses are palpable, then the clinician should assess for differentiating factors when considering limb ischemia, vascular pathologies, and Achenbach syndrome.

Kordzadeh’s review found that the most common patient demographics in Achenbach syndrome were female patients under the age of 60. Using the clinical picture, patient demographics, physical exam, and lack of significant risk factors for other vasculitis diagnoses, clinicians may reliably diagnose Achenbach syndrome without further investigations. If there are doubtful circumstances, the patient may undergo Doppler ultrasonography.^12^ This algorithmic approach offers a starting point from which a clinician may base their decisions. However, it is important to note that this algorithm has not been validated and is a theoretical decision tool based upon 12 case reports.

In summary, Achenbach syndrome is a benign and self-resolving disorder that usually presents as an atraumatic ecchymosis of a finger. The treatment is reassurance. Our patient was an otherwise healthy 54-year-old White female with a remote history of tobacco use and a normal ultrasound arterial duplex who underwent a hospital-to-hospital transfer by ambulance, was treated with a continuous infusion of medication and evaluated by multiple specialists, and underwent advanced imaging with the resultant diagnosis of Achenbach syndrome. Each intervention posed an additional risk and cost for the patient. Our hope is that by increasing awareness of this diagnosis, we may limit future potentially harmful interventions.

## CONCLUSION

It is essential for clinicians to be aware of the differential diagnosis of Achenbach syndrome. Once acute limb ischemia is ruled out, and the clinical picture is consistent, clinicians may practice good healthcare stewardship and limit invasive, potentially harmful, and expensive workup by reassuring patients of the benign nature of this condition.
